# Inhibition of DNA Repair Mechanisms and Induction of Apoptosis in Triple Negative Breast Cancer Cells Expressing the Human Herpesvirus 6 U94

**DOI:** 10.3390/cancers11071006

**Published:** 2019-07-18

**Authors:** Francesca Caccuri, Michele Sommariva, Stefania Marsico, Francesca Giordano, Alberto Zani, Arianna Giacomini, Cornel Fraefel, Andrea Balsari, Arnaldo Caruso

**Affiliations:** 1 Department of Molecular and Translational Medicine, University of Brescia, Brescia 25123, Italy; 2 Dipartimento di Scienze Biomediche per la Salute, Università degli Studi di Milano, Milan 20133, Italy; 3 Department of Pharmacy, Health and Nutritional Sciences, University of Calabria, Arcavacata di Rende, Cosenza 87036, Italy; 4 Institute of Virology, University of Zurich, Zurich 8057, Switzerland

**Keywords:** HHV-6 U94, HSV-1 amplicon vector, triple negative breast cancer cells, gene expression profile, DNA repair, cell cycle, apoptosis

## Abstract

Triple-negative breast cancer (TNBC) accounts for 15–20% of all breast cancers. In spite of initial good response to chemotherapy, the prognosis of TNBC remains poor and no effective specific targeted therapy is readily available. Recently, we demonstrated the ability of U94, the latency gene of human herpes virus 6 (HHV-6), to interfere with proliferation and with crucial steps of the metastatic cascade by using MDA-MB 231 TNBC breast cancer cell line. U94 expression was also associated with a partial mesenchymal-to-epithelial transition (MET) of cells, which displayed a less aggressive phenotype. In this study, we show the ability of U94 to exert its anticancer activity on three different TNBC cell lines by inhibiting DNA damage repair genes, cell cycle and eventually leading to cell death following activation of the intrinsic apoptotic pathway. Interestingly, we found that U94 acted synergistically with DNA-damaging drugs. Overall, we provide evidence that U94 is able to combat tumor cells with different mechanisms, thus attesting for the great potential of this molecule as a multi-target drug in cancer therapy.

## 1. Introduction

Breast cancer is the most frequently diagnosed malignancy and the second leading cause of tumor-related deaths in women worldwide [1,2,3]. Every year, nearly 1.7 million of breast cancer cases are diagnosed globally, and approximately 15–20% belong to the triple-negative (TNBC) subtype [4,5,6]. This family of cancer is negative for the expression of estrogen receptor (ER), progesterone receptor (PR), and human epidermal growth factor receptor 2 (HER2). As compared with hormone receptor-positive breast cancers, TNBCs are characterized by an extremely aggressive clinical course, younger age at onset, worse prognosis, great prevalence of highly proliferating grade tumors at diagnosis, enhanced metastatic potential, and poor clinical outcomes, as attested by the higher relapse and lower survival rates [7,8,9]. Almost 80–90% of these tumors are invasive ductal carcinomas, while the rest are classified as apocrine, lobular, adenoid cystic, medullary, and metaplastic [10]. Moreover, at diagnosis, these tumors are positive for lymph-vascular invasion and have already metastasized to lymph-nodes [11]. 

Due to the lack of ER, PR and HER2 receptors, TNBCs do not respond to hormone-based therapies, therefore chemotherapy still remains the standard of care treatment. The actual therapeutic approaches are represented by cytotoxic chemotherapy targeting DNA (i.e., platinum compounds), cell division (microtubule stabilizers, such as taxanes), and cell proliferation (anthracycline) [5,12]. However, an optimal chemotherapy regimen for TNBC therapy has to be established. Several other agents are being developed in the setting of managing TNBC including inhibitors of Poly (ADP-ribose) polymerase (PARP), angiogenesis, epidermal growth factor receptor (EGFR), Tyrosine kinases, mammalian target of rapamycin (mTOR), and statins [5,12,13]. 

Viruses represent a cancer cause and solution. Indeed, beside their role in promoting cancer development, in the last decades the virotherapy using oncolytic viruses has been proposed as a promising cancer therapy [14,15]. Some viruses, such as herpesviruses, express a handful of genes during latency that are known to manipulate different cell functions, particularly those affecting the life span and proliferative potential of the latently infected cells. In particular, HHV-6 expresses during latency U94, a nuclear targeting protein [16] that possesses DNA-binding, exonuclease and helicase-ATPase activities [17,18,19,20] and interacts with TATA-binding protein [19] suggesting a role for the viral protein in viral gene regulation and DNA replication.

Previous studies demonstrated that the stable expression of U94 suppressed transformation mediated by the oncogene H-ras in NIH 3T3 [21] and tumorigenesis of prostate cancer PC3 cell line [22]. Recently, we demonstrated the ability of the viral protein to interfere with the individual steps of the metastatic cascade by using MDA-MB 231 cell line [23]. Indeed, the expression of the viral protein delivered by a herpes simplex virus type-1-based amplicon vector (HSV-1) strongly inhibited cell migration, invasiveness and clonogenicity of breast cancer cells through the downmodulation of the proto-oncogene Src and its downstream signaling pathways. Interestingly, the block of the metastatic events was accompanied with a partial mesenchymal-to-epithelial transition (MET) and with the S phase inhibition of the cell cycle. All these data were confirmed in in vivo experiments in which we found that the viral protein exerted a long-term control of tumor growth, invasiveness and metastatization [23]. Fascinatingly, U94 was able to create a tumor microenvironment unfavorable to neo-vessels formation as demonstrated by the lack of blood vessels infiltrating the U94 positive tumors, an event inhibiting the supply of growth factors to the malignant cells [23]. Indeed, it has been demonstrated that the expression of the latency gene strongly inhibits angiogenesis and migration of blood and lymphatic human endothelial cells (ECs) in vitro and vasculogenesis ex vivo [16]. The role of U94 in favoring a less aggressive phenotype in cancer cells was confirmed on cervical cancer cells (HeLa) [23], whereas the antitumor capability of U94 was further demonstrated in glioma cell lines U251 and U87 [24,25].

The nuclear localization of the protein suggests that it might bind to DNA sequences and regulate transcription. In order to gain deep insight on the mechanisms of action of the viral protein, we characterized its molecular targets by performing comprehensive gene expression profile. In the current study, we demonstrate that U94 is able to induce apoptosis following downmodulation of DNA repair gene expression. Moreover, we identify U94 as a chemosensitizer molecule for TNBCs. 

## 2. Results

### 2.1. U94 Alters the Transcriptome Profile of MDA-MB 231 Cells 

**Figure 1:**
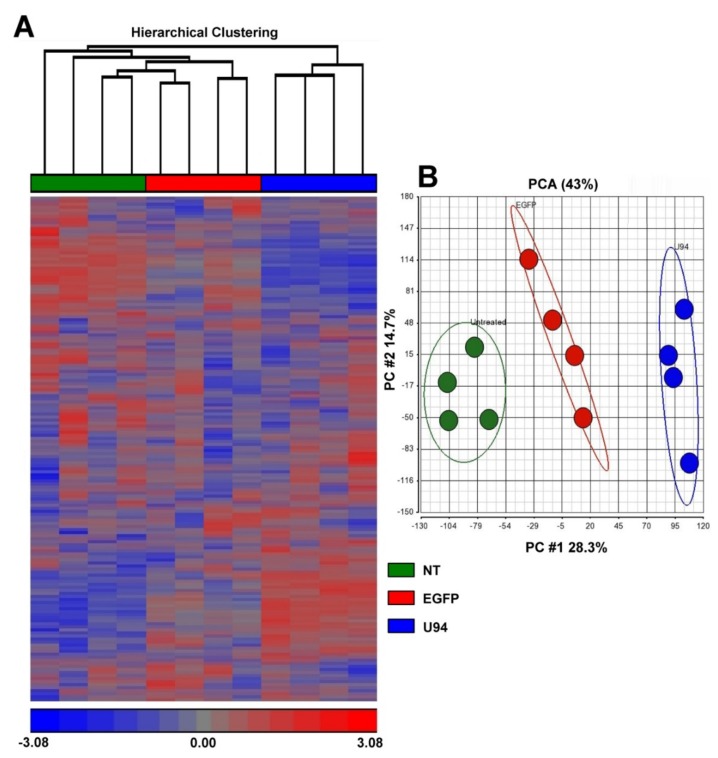
Transcriptional profile of MDA-MB 231 breast cancer cells expressing U94. (**A**) Unsupervised hierarchical clustering of MDA-MB 231 breast cancer cell line not treated (NT) (green), expressing EGFP (EGFP^+^) control (red) or expressing U94 (U94^+^) (blue) performed on 18537 genes obtained after normalization and filtering procedures (*n* = 4 samples/group). (**B**) Principal Component Analysis (PCA) conducted on 18537 genes obtained after normalization and filtering procedures (*n* = 4 samples/group) expressed by MDA-MB 231 cells NT (green), EGFP^+^ control (red) or U94^+^ (blue). Each dot represents a sample.

To better understand the mechanisms underlying the anti-proliferative effect of U94, we conducted a comprehensive gene expression analysis on U94-expressing (U94^+^) MDA-MB 231 breast cancer cells. We first applied a hierarchical clustering algorithm to analyze similarities among samples and among genes, using data obtained from the expression profiles of all samples. Figure 1A shows that samples - examined in quadruplicate to validate the reproducibility and reliability of our experimental procedure - clustered into three major groups on the basis of their expression profiles. Unsupervised hierarchical clustering revealed that not treated (NT) and EGFP-expressing (EGFP^+^) cells have a similar transcriptome profile, whereas U94^+^ cells segregated apart from the other two groups (Figure 1A). Moreover, Principal Component Analysis (PCA) [26] confirmed that U94^+^ cells exhibited a completely different gene expression profile, indicating that U94 profoundly impacted on cancer cell biology (Figure 1B). Among the 18537 genes available in our microarray platform, after normalization and filtering procedures, we found 2381 genes differentially expressed at False Discovery Rate (FDR) < 0.05 between U94^+^ and EGFP^+^ MDA-MB 231 cells. Specifically, 1523 gene were downmodulated, and 858 genes were upmodulated in U94^+^ cells as compared to EGFP^+^ cells (Supplementary Table S1).

### 2.2. U94 Induces Downregulation of Specific Gene sets in MDA-MB 231 Breast Cancer Cells

**Figure 2:**
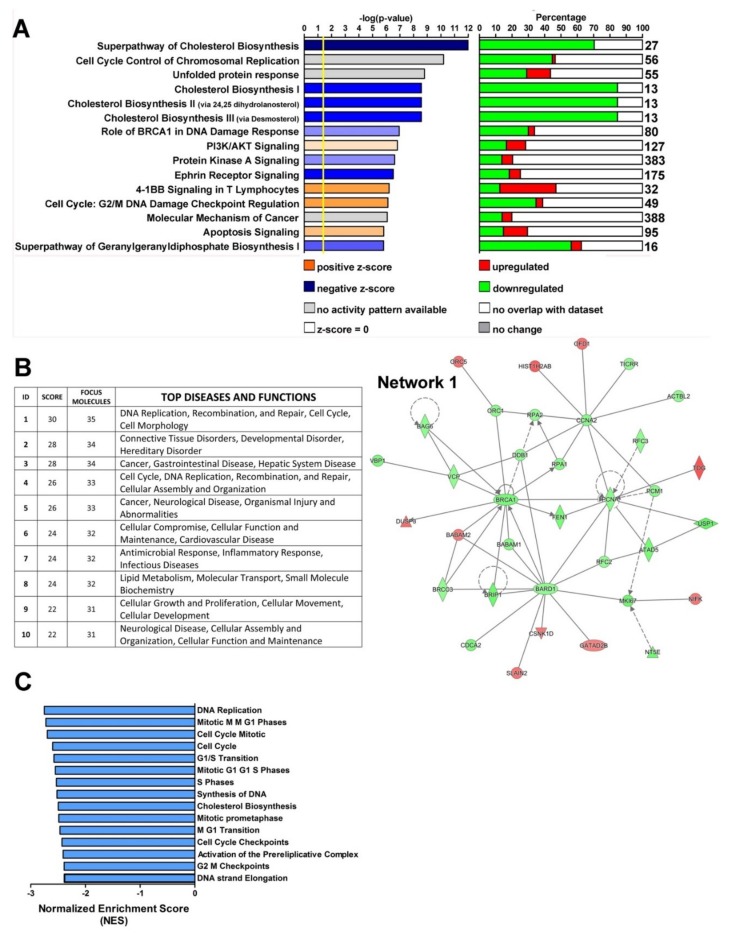
Functional analysis performed on differentially expressed genes in U94^+^ cells. (**A**) Top 15 canonical pathways differentially regulated between U94^+^ and EGFP^+^ MDA-MB 231 cells. These pathways are the results of Ingenuity Pathway analysis (IPA) “Core Analysis” conducted on 2381 genes with FDR < 0.05 differentially expressed between U94^+^ and EGFP^+^ MDA-MB 231 cells. Graph shows category scores. The yellow line (threshold) indicates the minimum significance level (scored as −log(*p*-value) from Fisher’s exact test). Stacked bar chart shows the percentage of genes downregulated (green), upregulated (red) and genes not overlapping with our data set (white) in each canonical pathway. The number at the top of each bar indicates the total number of genes present in the considered canonical pathway. (**B**) Top 10 molecular networks revealed by IPA analysis of 2381 genes with False Discovery Rate (FDR) < 0.05 differentially expressed between U94^+^ and EGFP^+^ MDA-MB 231 cells. (Right Panel) The most significant molecular network, “DNA Replication, Recombination and Repair, Cell Cycle, Cell Morphology”. Node color indicates up- (red) and downregulated genes (green). Node shapes represent functional classes of gene products. (**C**) Barplot of the Normalized Enrichment Score (NES) for the top 15 significant pathways identified by GSEAPreranked analysis comparing U94^+^ and EGFP^+^ MDA-MB 231 breast cancer cell line.

Functional analysis, performed by Ingenuity Pathways Analysis (IPA), of the 2381 genes with FDR < 0.05 revealed that pathways related to cell cycle progression and DNA damage response were downregulated in U94^+^ MDA-MB 231 cells as compared to EGFP^+^ cells (Figure 2A). Pathways associated with cholesterol biosynthesis also appeared to be influenced by U94 expression (Figure 2A). The ability of U94 in affecting cell cycle and DNA repair genes was also confirmed by network analysis since “DNA Replication, Recombination and Repair, Cell Cycle, Cell Morphology” was the network displaying the highest score (Figure 2B, left panel). To further corroborate our findings, we analyzed our microarray data by Gene Set Enrichment Analysis (GSEA) [27,28,29], performed to identify over- or under-represented pathways in U94^+^ compared to EGFP^+^. GSEA analysis showed that a total of 241 different gene sets reached the significance threshold of FDR < 0.25. 240/241 pathways were under-expressed in U94^+^ versus EGFP^+^ cells, indicating again that the U94 protein was able to switch-off the cellular machinery. Of note, the first 15 significantly enriched gene sets (all downmodulated in U94^+^ versus EGFP^+^ cells) were related to cell cycle progression and DNA replication (Figure 2C), confirming IPA analysis.

**Figure 3:**
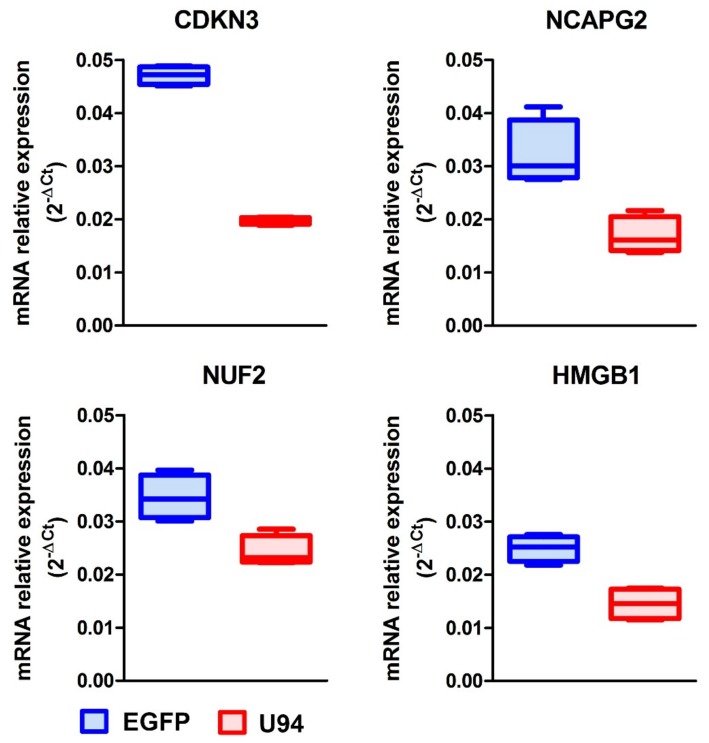
Validation of gene expression profile results by real-time PCR analysis. mRNA level of CDKN3, NCAPG2, NUF2 and HMGB1 was determined by real-time-PCR. Results are presented as 2^−ΔCt^.

In order to validate our microarray data, four representative genes were selected from the 1523 downmodulated gene list for their involvement in cell cycle regulation and DNA repair mechanisms. mRNA level was evaluated by real-time PCR analysis. In particular, we examined the expression level of the cyclin-dependent kinase inhibitor 3 (CDKN3), Non-SMC Condensin II Complex Subunit G2 (NCAPG2), Ndc80 kinetochore complex component (NUF2) and High Mobility Group Box 1 (HMGB1) genes, which are involved either in cancer cell proliferation or in mediating DNA repair processes. [30,31,32,33,34,35,36,37,38,39]. The real-time PCR analysis confirmed the microarray data (Figure 3). Indeed, the mRNA levels of CDKN3, NCAPG2, NUF2, and HMGB1 genes were found to be downregulated in U94^+^ cells, as compared to control (EGFP^+^) cells. Taken together, these data on one side further confirm our previous evidences on the ability of U94 to inhibit tumor cell proliferation causing the breast cancer cell cycle arrest [23] while, on the other side, they attest for the ability of U94 to interfere with the breast cancer cell DNA repair mechanisms leading to inhibition of their survival.

### 2.3. U94 Induces Breast Cancer Cell Apoptosis

**Figure 4:**
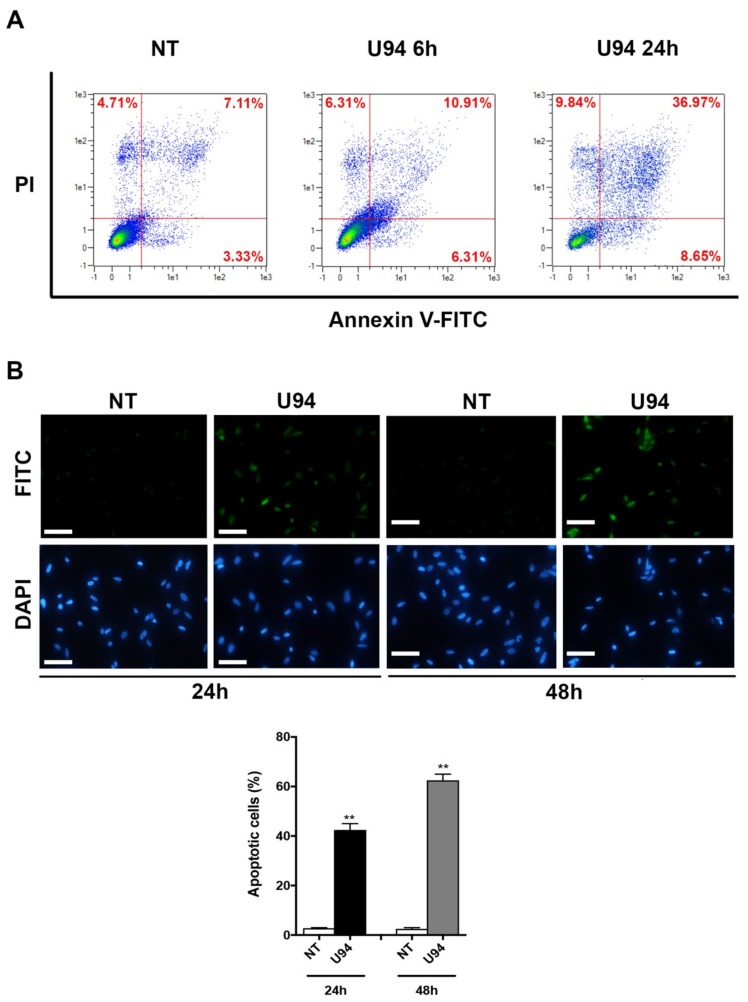
U94 expression in MDA-MB 231 cells induces apoptosis. (**A**) MDA-MB 231, 6 and 24 h post-transduction, were stained with Annexin V-FITC and PI. Apoptosis was assessed by flow cytometry. The four quadrants represent living cells (lower left), early apoptotic (lower right), late apoptosis (upper right) or necrotic (upper left) stages. Values represent the percentages of each quadrant. (**B**) Terminal deoxynucleotidyl transferase-mediated dUTP Nick End Labeling (TUNEL) staining in MDA-MB 231 cells transduced or not with amplicons for 24 and 48 h. DAPI staining was used to visualize the cell nucleus. The histograms represent the mean ± SD of apoptotic cells from two independent experiments performed in triplicate. Statistical analysis was performed by unpaired Student’s *t*-test, ** *p* < 0.01. Scale bar: 100 µm.

Induction of DNA damage following block of repair mechanisms triggers apoptosis. The main morphological changes of the apoptotic process are nuclear shrinkage, chromatin condensation [40] and anomalies in structure and stability of the cell membrane components [41]. In particular, the translocation of phosphatidylserine (PS) from the inner leaflet of the plasma membrane to the cell surface and the increased permeability of the membrane are characteristics of apoptotic cells [42]. Morphological changes on cell membranes of U94^+^ cells were analyzed at 6 and 24 h post-transduction, by staining cells with a combination of fluorescently labeled Annexin V and PI, which discriminates viable cells (Annexin V^−^/PI^−^), early apoptotic cells (Annexin V^+^/PI^−^), late apoptotic cells (Annexin V^+^/PI^+^) and necrotic cells (Annexin V^−^/PI^+^). Flow cytometric analysis revealed that following U94 expression, breast cancer cells undergo apoptosis. As shown in Figure 4A, the percentage of apoptotic cells was 17.22% at 6 h and increased to 45.62% at 24 h post-transduction, whereas the percentage of apoptotic cells at baseline was 10.44% only. In particular, the percentages of early apoptotic cells (lower right quadrant) shifted from 3.33% in NT cells to 6.31% and 8.65%, at 6 and 24 h post-transduction respectively, while the percentages of late apoptotic cells (upper right quadrant) increased from 7.11% in NT cells to 10.91% and 36.97% at 6 and 24 h post-transduction, respectively. Thereafter, we determined changes in the internucleosomal fragmentation profile of genomic DNA by terminal deoxynucleotidyl transferase dUTP nick end labeling (TUNEL) assay which detects the DNA breaks occurring during the latest stages of apoptosis. As shown in Figure 4B, U94^+^ cells were TUNEL^+^ (FITC^+^), whereas NT cells were found to be TUNEL^−^. Taken together, our data demonstrate that the expression of U94 triggers breast cancer cells to undergo apoptosis.

### 2.4. Activation of the Intrinsic Apoptotic Pathway by U94 Expression

**Figure 5:**
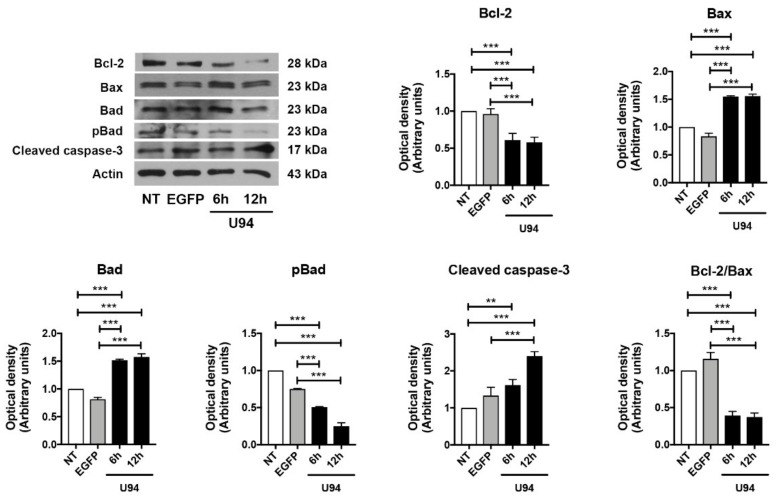
U94 induces apoptosis through the mitochondrial pathway. Immunoblots of Bcl-2, Bax, Bad, pBad and cleaved caspase-3 protein levels in MDA-MB 231 cells transduced or not with amplicons for 6 and 12 h. The histograms represent the mean ± SD of three separate experiments in which band intensities were evaluated as optical density (OD) and expressed as fold change versus control samples. Data were analyzed for statistical significance by One-way ANOVA followed by Tukey’s *post hoc* test. ** *p* < 0.01 and *** *p* < 0.001 versus control samples.

**Figure 6:**
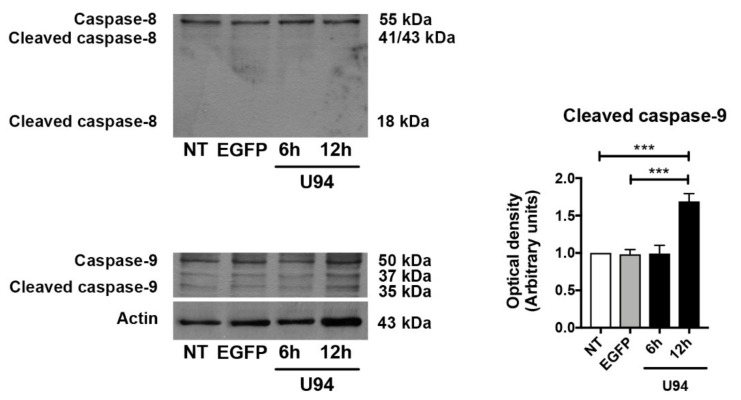
U94 induces intrinsic apoptosis. Immunoblots of caspase-8, cleaved caspase-8, caspase-9 and cleaved caspase-9 protein levels in MDA-MB 231 cells transduced or not with amplicons for 6 and 12 h. The histograms represent the mean ± SD of three separate experiments in which band intensities were evaluated as optical density (OD) and expressed as fold change versus control samples. Data were analyzed for statistical significance by One-way ANOVA followed by Tukey’s *post hoc* test. *** *p* < 0.001 versus control samples.

**Figure 7:**
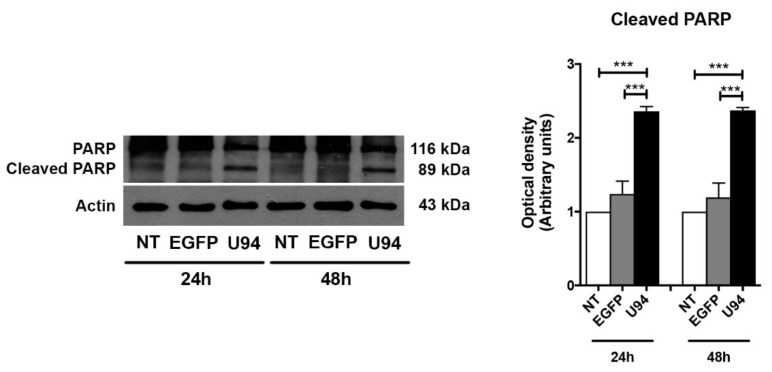
Late apoptosis in U94 expressing cells. Immunoblots of poly (ADP-ribose) polymerase (PARP) protein levels in MDA-MB 231 cells transduced or not with amplicons for 24 and 48 h. The histograms represent the mean ± SD of three separate experiments in which band intensities were evaluated as optical density (OD) and expressed as fold change vs. control samples. Data were analyzed for statistical significance by One-way ANOVA followed by Tukey’s *post hoc* test. *** *p* < 0.001 versus control samples.

The pathway involved in cell death can be resumed into few critical proteins. In particular, the Bcl-2 family of proteins controls a critical step in commitment to apoptosis by regulating permeabilization of the mitochondrial outer membrane. This family includes proteins which either promote or inhibit apoptosis that are divided into three groups based on their primary function: antiapoptotic proteins such as Bcl-2, proapoptotic pore-formers such as Bax and proapoptotic BH3-only proteins such as Bad [43]. Thus, the expression levels of these key apoptotic markers in MDA-MB 231 cells upon U94 expression was evaluated by immunoblotting. As shown in Figure 5, the expression level of Bcl-2 strongly decreased in U94^+^ cells both at 6 h and 12 h post-transduction as compared to control cells, whereas a concomitant increase of Bax and Bad was observed. As a consequence, the Bcl-2/Bax ratio decreased at both time points upon the viral protein expression. Since Bad can be phosphorylated and this phosphorylation converts the proapoptotic Bad into an antiapoptotic protein [44], we examined its phosphorylation status upon U94 expression. As expected, U94^+^ cells displayed increased levels of Bad compared to its phosphorylated form either at 6 or 12 h post-transduction (Figure 5, Figures S1 and S4). The Bcl-2 family controls cell death primarily by regulating the mitochondrial outer membrane permeabilization, thus leading to the release of intermembrane space proteins, caspase activation and apoptosis [43]. We analyzed the expression levels of the executioner caspase-3, which has no activity until it is cleaved by an initiator caspase upon apoptotic signaling events. As expected, the expression of the active effector caspase-3 in its cleaved form was found to be increased in U94^+^ cells at 12 h post-transduction, as compared to control cells (Figure 5, Figures S1 and S4). Caspase-3 is activated in both extrinsic (death ligand) and intrinsic (mitochondrial) apoptotic pathways and its processing and activation can be mediated by the executioner caspase-8 or -9, respectively [45,46,47]. To gain deep insight into the mechanisms by which U94 induces apoptosis, we evaluated the levels of the extrinsic and intrinsic apoptotic markers caspase-8 and caspase-9. As shown in Figure 6 (Figures S2 and S4), caspase-8 was found to be present in its inactive (not cleaved) form in both control and U94^+^ cells. On the contrary, caspase-9 in its active cleaved form, was found to be significantly higher in U94^+^ cells at 12 h post-transduction as compared to control cells, attesting for the involvement of the intrinsic apoptotic pathway in U94 activity. Activation of effector caspases such as caspase-3 leads to downstream cleavage of various cytoplasmic or nuclear substrates including poly (ADP-ribose) polymerase (PARP), which is involved in DNA repair mechanisms and apoptosis [48]. PARP inactivation prevents depletion of NAD and ATP, which are required for later events in apoptosis [49]. We evaluated the proteolysis of PARP and found that the expression level of cleaved PARP strongly increased in U94^+^ cells as compared to control cells (Figure 7A, Figures S3 and S4). Altogether, our data indicate that the viral protein induces activation of the caspase-dependent intrinsic apoptotic pathway in MDA-MB 231 cells.

### 2.5. Synergism of U94 and DNA-Damaging Drugs against Breast Cancer Cells

**Figure 8:**
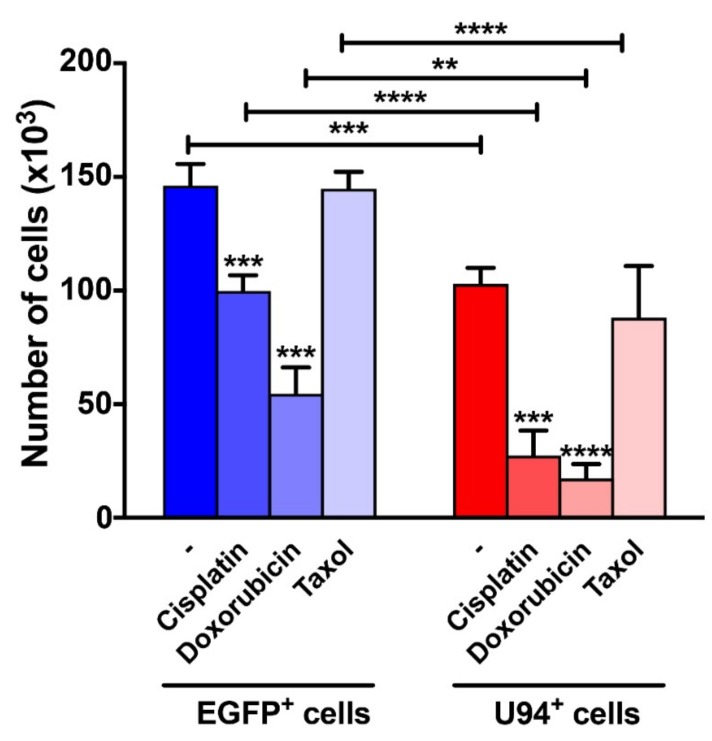
U94 sensitizes cancer cells to DNA-damaging drugs. MDA-MB 231 cells expressing either EGFP (blue bars) or U94 (red bars) were exposed to 100 µM of cisplatin, 10 µM of doxorubicin or 50 µM of docetaxel. Cell proliferation was determined using an automatic cell counter. Values are reported as mean ± SEM of four replicates. ** *p* < 0.01, *** *p* < 0.001 and **** *p* < 0.0001 by Two-way ANOVA followed by Sidak’s multiple comparison test.

Impaired DNA repair machinery is likely to increase cancer cell killing by DNA-damaging chemotherapeutic drugs [50,51]. Therefore, we explored the possibility that U94 might act as a chemo-sensitizer molecule. To translate our findings from molecular to cellular level, MDA-MB 231 cells expressing or not expressing U94 were exposed to cisplatin and doxorubicin, two known DNA-damaging drugs [52,53]. As shown in Figure 8, in agreement with our previous findings [23], U94 is *per se* able to reduce breast cancer cell proliferation (EGFP^+^ vs. U94^+^ cells [Mean ± SEM]: 146,182 ± 4758 vs. 103,012 ± 3484, *p* = 0.0005). Interestingly, U94^+^ cells exhibited a drastic decrease in cell growth upon cisplatin or doxorubicin treatment as compared to control cells (cisplatin-treated EGFP^+^ vs. U94^+^ cells [Mean ± SEM]: 99,801 ± 3464 vs. 27,459 ± 5488, *p* < 0.0001; and doxorubicin-treated EGFP^+^ vs. U94^+^ cells [Mean ± SEM]: 54,597 ± 5781 vs. 17,248 ± 3216, *p* = 0.0031). The increased efficiency of U94^+^ cisplatin vs. cisplatin and of U94^+^ doxorubicin vs doxorubicin was 49.5% and 25.6% respectively. This finding attests for the ability of U94 to render MDA-MB 231 cells more sensitive to the activity of DNA-damaging drugs. At the same time, U94 did not show any synergistic effect with taxol, a microtubule inhibitor [54] (taxol-untreated vs. taxol-treated U94^+^ cells [Mean ± SEM]: 103,012 ± 3484 vs. 88,147 ± 11,330, *p* = 0.9003). Indeed, the differences in cell proliferation observed upon taxol treatment EGFP^+^ vs U94^+^ cells ([Mean ± SEM]: 144,820 ± 3705 vs. 88,147 ± 1133, *p* < 0.0001) are merely related to the ability of U94 to interfere with MDA-MB 231 cell growth. Collectively, these data suggest that U94 makes cancer cells more susceptible to DNA damaging drugs.

### 2.6. U94 Induces Apoptosis in Different TNBCs

**Figure 9:**
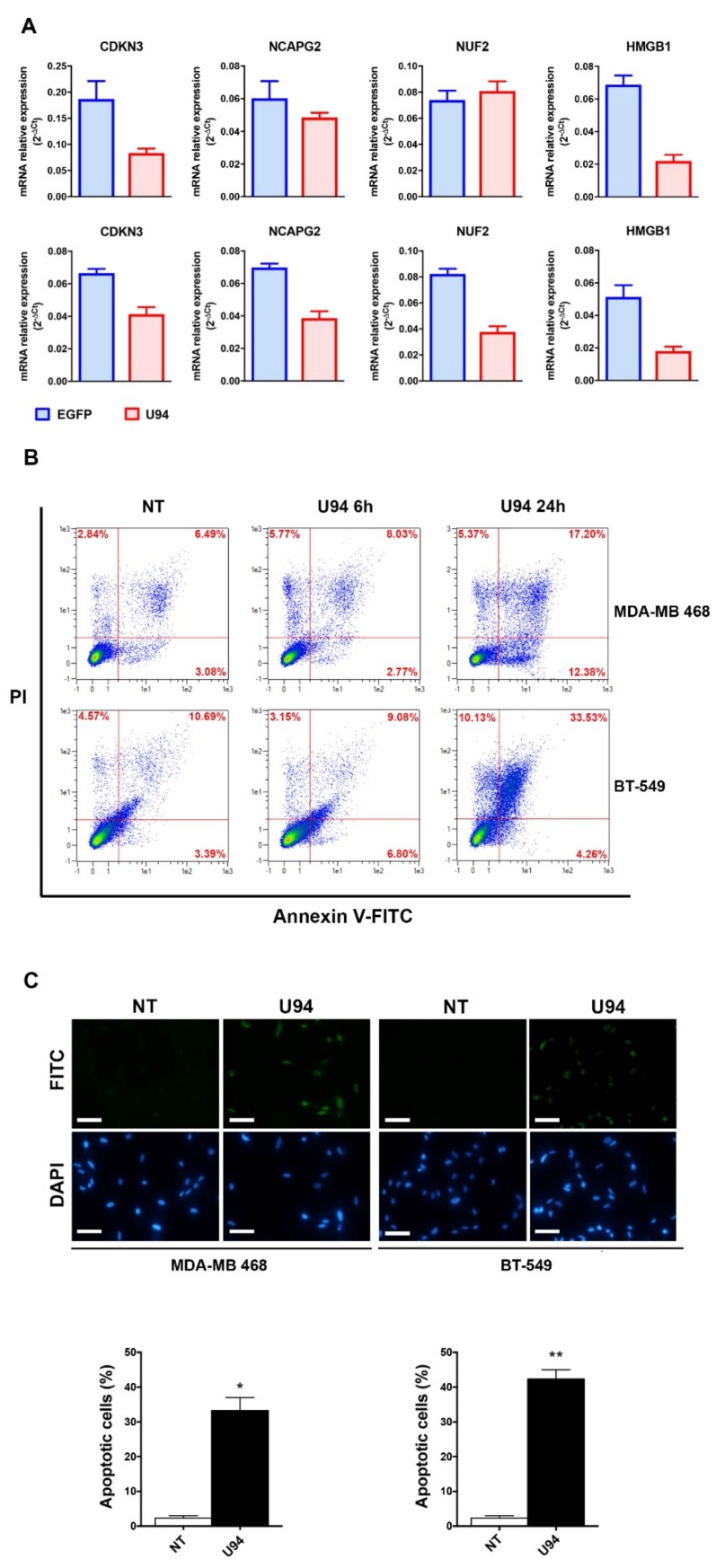
U94 induces cell cycle arrest, inhibition of DNA repair and apoptosis in MDA-MB 468 and BT-549 cell lines. (**A**) mRNA level of CDKN3, NCAPG2, NUF2 and HMGB1 was determined by real-time-PCR in MDA-MB 468 cells (upper panel) and BT-549 (lower panel). (**B**) MDA-MB 468 and BT-549 cells, 6 and 24 h post-transduction, were stained with Annexin V-FITC and PI. Apoptosis was assessed by flow cytometry. The four quadrants represent living cells (lower left), early apoptotic (lower right), late apoptosis (upper right) or necrotic (upper left) stages. Values represent the percentages of each quadrant. (**C**) Terminal deoxynucleotidyl transferase-mediated dUTP Nick End Labeling (TUNEL) staining in MDA-MB 468 and BT-549 cells transduced or not with amplicons for 24 h. DAPI staining was used to visualize the cell nucleus. The histograms represent the mean ± SD of apoptotic cells from two independent experiments performed in triplicate. Statistical analysis was performed by unpaired Student’s *t*-test, * *p* < 0.05 and ** *p* < 0.01. Scale bar: 100 µm.

The ability of U94 to affect cell cycle and DNA repair genes was also evaluated in MDA-MB 468 and in BT-549 TNBC cell lines, by real-time PCR. The experiments were carried-out at 24 h post-transduction with EGFP- and U94-expressing plasmids. As shown in Figure 9A, the mRNA levels of CDKN3, NCAPG2, and HMGB1 genes were found to be downregulated in both MDA-MB 468 and BT-549 U94^+^ cells, as compared to control (EGFP^+^) cells and NUF2 in BT-549 cells. These data further confirm the ability of U94 to inhibit tumor cell proliferation by interfering with breast cancer cell DNA repair mechanisms. To gain deeper insight on the ability of U94 to induce apoptosis in MDA-MB 468 and BT-549 cells, we performed the Annexin V/PI and the TUNEL assays. Morphological changes on cell membranes were analyzed at 6 and 24 h post-transduction with the U94-expressing plasmid, by staining cells with a combination of fluorescently labeled Annexin V and PI. Flow cytometric analysis revealed that following U94 expression, MDA-MB 468 and BT-549 cells undergo apoptosis. As shown in Figure 9B, the percentage of apoptotic MDA-MB 468 cells was 10.8% at 6 h and increased to 29.58% at 24 h post-transduction, whereas the percentage of apoptotic cells at baseline was only 9.57%. The percentage of apoptotic BT-549 cells was 15.88% at 6 h and increased to 37.79% at 24 h post-transduction, whereas the percentage of apoptotic cells at baseline was only 14.08%. MDA-MB 468 U94^+^ cells were found to be in the early apoptotic stage at 6 h and progressed to late apoptosis at 24 h post-transduction. In particular, in MDA-MB 468 cells the percentages of early apoptotic cells (lower right quadrant) shifted from 3.08% in NT cells to 2.77% and 12.38%, at 6 and 24 h post-transduction respectively, while the percentages of late apoptotic cells (upper right quadrant) increased from 6.49% in NT cells to 8.03% and 17.20% at 6 and 24 h post-transduction, respectively. Instead, in BT-549 the percentages of early apoptotic cells (lower right quadrant) shifted from 3.39% in NT cells to 6.80% and 4.26%, at 6 and 24 h post-transduction respectively, while the percentages of late apoptotic cells (upper right quadrant) changed from 10.69% in NT cells to 9.08% and 33.53% at 6 and 24 h post-transduction, respectively. To further confirm the induction of apoptosis in MDA-MB 468 and BT-549 cells, the DNA breaks occurring during the latest stages of apoptosis were evaluated by TUNEL assay. As shown in Figure 9C, U94^+^ cells were TUNEL^+^ (FITC^+^), whereas NT cells were found to be TUNEL^−^ in both MDA-MB 468 and BT-549 cells. Taken together, these data demonstrate that the expression of U94 triggers apoptosis in different TNBCs.

## 3. Discussion

Standard chemotherapy for cancer aims to produce replication stress-induced DNA damage thereby promoting cell death. The ability of cancer cells to recognize this damage and initiate DNA repair is an important mechanism for drug resistance and poor therapeutic efficacy [55]. As a consequence, new approaches employing novel compounds and different mechanisms are needed. As such, the possibility of supporting the current anticancer therapies by making tumors more susceptible to inhibitors of DNA repair mechanisms may represent an interesting outcome for developing new anticancer approaches. 

Lack of apoptosis is considered as one of the symptoms of tumorigenicity [56], whereas the induction of apoptosis represents a key target for cancer therapy [57]. Our data highlight the ability of U94 to exert anticancer activity on TNBC by inhibiting DNA damage repair genes, cell cycle and eventually leading to intrinsic apoptotic cell death. Indeed, U94 was found to downregulate the antiapoptotic marker Bcl-2 and upregulate the expression of proapoptotic markers, such as Bax and Bad, leading to caspase-9 and PARP cleavage. Interestingly, the viral protein was found to operate synergistically with DNA-damaging drugs but not with microtubule inhibitors. This cooperation occurs by different mechanisms. DNA-damaging drugs operate by directly targeting to DNA. For instance, doxorubicin is able to intercalate the DNA via its insertion between the planar DNA base pairs [58] whereas cisplatin binds to and cross-links DNA which affects DNA strand synthesis and replication [59]. On the other hand, U94 is able to inhibit the DNA repair mechanisms at gene level by downmodulating the transcription of genes specifically involved in DNA damage repair. It has been demonstrated that U94 is a nuclear targeting protein [16], therefore it is more likely to hypothesize that it exerts its activity in the nucleus. Further studies will be necessary to identify the nuclear targets, if any, of the viral protein. The relevance of U94 as a therapeutic molecule against TNBC resides in the evidence of its cancer cell killing effect in the absence of toxicity on normal cells, such as human primary endothelial cells [16]. In light of our evidence, U94 can be considered a DNA damage response (DDR) inhibitor. The DNA repair is strictly linked to the DDR, which involves cellular mechanism that safeguards against DNA damage and that has been recognized as an important innate tumor suppressor pathway [60,61]. It is worth noting that cancers deficient in DNA mismatch repair display a favorable prognosis [62]. Tumors carrying a high number of mutations respond well to immunotherapy [63,64,65] and the inhibition of DNA repair induces neo-antigens generation and impairs tumor growth [66]. Recent data showed that the permanent block of specific tumor suppressors involved in DNA repair might be applied for developing new therapeutic approaches [66]. In this context, it is likely that a continuous activity of U94 in the inhibition of DNA repair processes in immunocompetent individuals may well trigger a long-lasting immune surveillance leading to a better disease outcome for patients.

Overall, the most interesting aspect of U94 is that its activity is carried out by assaulting tumor cells from different sides and with different mechanisms of action. Indeed, our previous study demonstrated that U94 is able to induce a transient blockage of cell cycle and proliferation together with a partial mesenchymal to epithelial transition (MET) of MDA-MB 231 cells. Interestingly, these effects were also evident in vivo [23]. Indeed, U94^+^ MDA-MB 231 cells generated very small and compact tumor masses which lack blood vessel infiltration, and have no ability to invade the surrounding adipose tissue compared to control, U94^−^ cells. Moreover, compared to U94^−^ tumors, the U94^+^ ones were characterized by a more differentiated phenotype. This evidence suggests that those MDA-MB 231 cells which do not respond to the U94-induced DNA damage and apoptosis undergo a MET and regress to a less aggressive phenotype. However, the possibility that cells with low proliferative capacity and refractory to U94 effects cannot be ruled out. Altogether, this knowledge led to hypothesize that cells resistant to the inhibition of DNA repair induced by U94 are likely to acquire proliferative disadvantage as compared to control cells.

Our microarray data also highlighted the ability of U94 to block the cholesterol biosynthesis pathway. The impact of cholesterol on oncogenic events, such as tumor development, cell migration and angiogenesis has been widely recognized [67,68] and, as a consequence, targeting cholesterol pathways is now considered a potential powerful strategy for cancer therapy [68]. In this context, the ability of U94 to target the cholesterol synthesis in tumor cells acquires a fundamental role, which deserves to be deeply investigated. The ability of U94 to interfere with cancer cell proliferation and, at the same time, to promote apoptosis points to the evidence that the viral protein acts as a cell killer in two ways on one side, by blocking DNA repair and, on the other side, by inhibiting the cholesterol synthesis.

U94 is also known to inhibit angiogenesis and lymphangiogenesis by stimulating the release of a soluble factor(s) [23]. Recently, we demonstrated that U94 induces upmodulation and release of HLA-G in ECs, and that this remodulation is directly related to the inhibition of angiogenetic properties observed in ECs upon U94 expression [69].

In conclusion, the ability of U94 to attack tumor cells with different mechanisms attests for the great potential of this molecule as anticancer agent. U94 showed to be a potential chemotherapy sensitizer for the selective killing of cancer cells that are deficient in DNA repair. Due to its multi-target activity, U94 represents a promising therapeutic TNBC treatment as a single agent or in combination.

## 4. Materials and Methods

### 4.1. Cell Cultures

Human breast cancer cells (MDA-MB 231, MDA-MB 468 and BT-549) were obtained from the American Type Culture Collection (ATCC) and grown according to ATCC product sheet recommendations.

### 4.2. Cell Transduction

The U94-expressing herpes simplex type-1 (HSV-1) amplicon vectors were constructed, produced and titrated as previously described [23]. Transduction of tumor cells was performed by incubating cells for 3 h in serum-free medium containing or not U94 or EGFP-expressing amplicons at multiplicity of infection (MOI) 1. Cells were then washed and incubated in complete medium. Transduction efficiency was assessed by flow cytometry.

In specific experiments, such as the Annexin V and the TUNNEL assay, cells were transduced with an amplicon vector expressing U94 only to avoid fluorescence interferences.

### 4.3. Reagents

Cisplatin, doxorubicin and taxol were purchased from Accord Healthcare Italia s.r.l. (Milan, Italy).

### 4.4. Gene Expression Profile and Bioinformatic Analysis

Not treated (NT), EGFP-expressing (EGFP^+^) and U94-expressing (U94^+^) cells (four replicates for each experimental condition) were subjected to microarray analysis to compare their gene expression profiles. Gene expression profiles were performed by the Integrated Biology Platform of Applied Research and Technology Development, Fondazione IRCCS Istituto Nazionale dei Tumori di Milano (Milan, Italy). Twenty-four h post-transduction, mRNA was isolated using the RNeasy Mini Kit (Qiagen, Valencia, CA, USA) according to manufacturer’s instructions. After quality check and quantification by 4200TapeStation (Agilent Technologies, Santa Clara, CA, USA) and Qubit Fluorometer (ThermoFisher Scientific, Waltham, MA, USA), respectively, RNA expression was assessed using the human Affymetrix Clariom S (Affymetrix ThermoFisher Scientific). Specifically, 100 ng of total RNA was used to generate the single-stranded cDNA samples for hybridization. Then, cDNA was enzymatically fragmented and biotinylated using the WT Terminal Labeling kit (ThermoFisher), combined with the hybridization buffer, and injected into the Human Clariom S arrays (Affymetrix ThermoFisher Scientific) targeting >20,000 well-annotated genes. Arrays were stained using the Affymetrix® GeneChip® Fluidics Station 450 and scanned with the 7G Affymetrix® GeneChip® Scanner 3000 (Affymetrix ThermoFisher Scientific). Raw data were pre-processed using RMA (Irizarry rma) as implemented in the oligo package of Bioconductor. Normalized data were collapsed from probe- to gene-level using the collapseRows function [70] of the WGCNA R package, selecting for each gene the probe with highest variation across samples [71]. The final dataset used in the present study included expression data of 18537 unique genes. Hierarchical clustering and Principal Component Analysis (PCA) analyses were performed using Partek software (Partek Inc., St. Louis, MO, USA).

Class comparison between U94^+^ and EGFP^+^ transduced cells was performed using log_2_ normalized data and limma R package [72]. Genes were considered statistically significant at a false-discovery rate (FDR) < 0.05. Pathways and networks significantly regulated in gene expression upon U94 expression were examined using the Core Analysis function included in Ingenuity Pathway Analysis (IPA, Qiagen Redwood City, CA, USA).

Pre-ranked Gene set enrichment analysis (GSEAPreranked) using javaGSEA Desktop Application v3.0 (http://www.broadinstitute.org/gsea/index.jsp) [28] was performed for functional analysis of our dataset. Genes were ranked according to the t test value retrieved from limma analysis. Pathways with FDR < 0.25 and nominal *p* value < 0.01 were considered statistically significant.

### 4.5. Real-Time PCR

The mRNA expression level of CDKN3, NCAPG2, NUF2 and HMGB1 was evaluated by Real-time PCR on the same stored frozen RNA used for microarray analysis. Total RNA was reverse-transcribed using the High-Capacity RNA-to-cDNA™ Kit (ThermoFisher Scientific). Real-time PCR was performed using TaqMan® Fast Universal PCR Master Mix (Applied Biosystems-Thermo Fisher Scientific) and StepOnePlus™ Real-Time PCR System (ThermoFisher Scientific), with the following TaqMan® gene expression assays (Applied Biosystems-Thermo Fisher Scientific) CDKN3 (Hs00193192_m1), NCAPG2 (Hs00914667_m1), NUF2 (Hs00230097_m1) and HMGB1 (Hs01923466_g1). The expression of each gene was normalized to β2M gene (Hs99999907_m1). Results are presented as 2^−ΔCt^, as previously reported [73].

### 4.6. Annexin V Assay

Annexin V assay is based on the detection of phosphatidylserine on the outer leaflet of the plasma membrane of cells, a characteristic of early apoptosis. Annexin assay was conducted using Annexin V-FITC Apoptosis Detection kit (Calbiochem, San Diego, CA, USA) and performed on cells transducted or not with different amplicons at different time points according to the manufacturer’s instructions. Briefly, after transduction cells were harvested and washed twice in cold PBS and resuspended at a concentration of 10^6^ cells/ml in 1× binding buffer. An aliquot of 500 μL was incubated with 1.25 μL Annexin V-FITC for 15 min in the dark at room temperature. Then, cells were centrifuged and resuspended in 500 μL of 1× binding buffer 10 µL of Propidium Iodide were added to each sample, and the fluorescein-5-isothiocyanate (FITC) and PI fluorescence were measured using flow cytometry through FL-1 filter (530 nm) and FL-2 filter (585 nm). Viable cells were annexin V negative and PI negative, early apoptotic cells were annexin V positive and PI negative, and late apoptotic cells or necrotic cells were annexin V positive and PI positive. Cells were analyzed by flow cytometry (FACScan, Becton Dickinson, San Jose, CA, USA) according to the manufacturer instructions. 

### 4.7. Western Blot Analysis

MDA-MB 231cells (2 × 10^6^) were transducted or not with different amplicons and cultured for 6, 12, 24 and 48 h. Then, cells were lysed in 500 μL of 50 mM Tris–HCl, 150 mM NaCl, 1% NP-40, 0.5% sodium deoxycholate, 2 mM sodium fluoride, 2 mM EDTA, 0.1% SDS, containing a mixture of protease inhibitors (aprotinin, phenylmethylsulfonyl fluoride, and sodium orthovanadate; Sigma-Aldrich, St. Louis, MO, USA) for total protein extraction. Equal amounts of proteins were resolved on 8 or 12% SDS-polyacrylamide gel, transferred to a nitrocellulose membrane and probed with, Poly (ADP-ribose) polymerase (PARP), Bcl-2, Bax, Bad, pBad, caspase-3, caspase-8 and caspase-9 specific antibodies (Santa Cruz, Biotechnology, CA, USA). To ensure equal loading and transfer, all membranes were stripped and incubated with anti-Actin antibody (Santa Cruz Biotechnology). The antigen-antibody complex was detected by incubation of the membranes with peroxidase-coupled goat anti-mouse or goat anti-rabbit antibodies and revealed using the ECL System (Amersham Pharmacia, Buckinghamshire, UK).

### 4.8. Terminal Deoxynucleotidyl Transferase dUTP Nick End Labeling (TUNEL) Assay

Apoptosis was determined by enzymatic labeling of DNA strand breaks using terminal deoxynucleotidyl transferase-mediated deoxyuridine triphosphate nick end-labeling (TUNEL). TUNEL labeling was conducted using APO-BrdU™ TUNEL Assay Kit (Promega, Madison, WI, USA) and performed according to the manufacturer’s instructions. Briefly, cells transduced or not with the amplicon for 24 and 48 h, were fixed in freshly prepared 4% paraformaldehyde solution in PBS (pH 7.4) for 25 min at 4 °C. After fixation, cells were permeabilized in 0.2% Triton® X-100 solution in PBS for 5 min. After washing twice with washing buffer for 5 min, the cells were covered with 100 μL of equilibration buffer at room temperature for 5–10 min. The labeling reaction was performed using terminal deoxynucleotidyl transferase end-labeling TdT and fluorescein-dUTP cocktail for each sample and incubated for 1 h at 37 °C where TdT catalyzes the binding of fluorescein-dUTP to free 3′OH ends in the nicked DNA. After rinsing, cells were washed with 20× SSC solution buffer and subsequently incubated with 100 μL of 4′,6-diamidino-2-phenylindole (DAPI; Sigma) to stain nuclei, protected from light, analyzed and photographed by using a fluorescence microscope (20× objective).

### 4.9. Chemotherapeutic Drugs Cytotoxic Activity on MDA-MB 231 Cell Line Upon U94 Expression

MDA-MB 231 breast cancer cell line was seeded at a density of 1.75 × 10^5^ cells in a 24-well plate. Twenty-four hours after seeding, cells were transduced as described above. Cytotoxicity was assessed by growth inhibition assay after a 24 h exposure to cisplatin (100 μM), doxorubicin (10 μM) or taxol (50 μM). Each experimental sample was run in quadruplicate. At the end of treatment, each well was washed with PBS to remove dead cells and debris. The remaining cells were detached from plate using Trypsin/EDTA (Corning, New York, NY, USA), resuspended in ISOTON® II Diluent (Beckman Coulter Life Sciences, Indianapolis, Indiana, IN, USA) and counted using Z2™ COULTER COUNTER® Analyzer (Beckman Coulter Life Sciences).

### 4.10. Statistical Analysis

Data obtained from multiple independent experiments are expressed as the mean ± standard deviation (SD) or standard error of the mean (SEM). Principal Component Analysis (PCA) was applied. Data were analyzed for statistical significance using Fisher’s exact test, One-way ANOVA followed by Tukey’s post hoc test or Two-way ANOVA followed by Sidak’s multiple comparison test. Differences were considered significant when *p* < 0.05. Statistical tests were performed using GraphPad Prism software (GraphPad Software, La Jolla, CA, USA).

## 5. Conclusions

The emergence of refractory cancer, in particular of TNBC, against the current anticancer drugs is worrying. To overcome the resistance and toxicity issues of current anti-cancer therapies, newer strategies need to be adopted. In this setting, U94 represents a promising therapeutic TNBC treatment as a single agent or in combination. It is a potential chemotherapy sensitizer for the selective killing of cancer cells that are deficient in DNA repair.

## Supplementary Materials

The following are available online at https://www.mdpi.com/2072-6694/11/7/1006/s1, Figure S1: Immunoblots of Bcl-2, Bax, Bad, pBad, Cleaved Caspase 3 and Actin protein levels in MDA-MB 231 cells, Figure S2: Immunoblots of Caspase 8, Cleaved Caspase 8, Caspase 9, Cleaved Caspase 9 and Actin protein levels in MDA-MB 231 cells, Figure S3: Immunoblots of PARP and Actin protein levels in MDA-MB 231 cells, Figure S4: Densitometry readings/intensity ratio of each band. Table S1: List of down- and up- modulated genes between U94 and EGFP transduced cells determined by class comparison, using limma R package, performed on log2 normalized microarray data.
