# Supplementary material for: Inhibition of DNA Repair Mechanisms and Induction of Apoptosis in Triple Negative Breast Cancer Cells Expressing the Human Herpesvirus 6 U94
Source: Cancers (Basel). 2019 Jul 18;11(7):1006. doi: 10.3390/cancers11071006 (PMC6679437; doi:10.3390/cancers11071006)
Supplement: Supplementary file 1 [file cancers-11-01006-s001.zip › cancers-530876-suppl/cancers-530876-suppl.pdf]

Supplementary Meterials: Inhibition of DNA repair mechanisms and induction of apoptosis in triple negative breast cancer cells expressing the human herpesvirus 6 U94

F. Caccuri, M. Sommariva, S. Marsico, F. Giordano, A. Zani, A. Giacomini, C. Fraefel, A. Balsari, A. Caruso

Figure 5

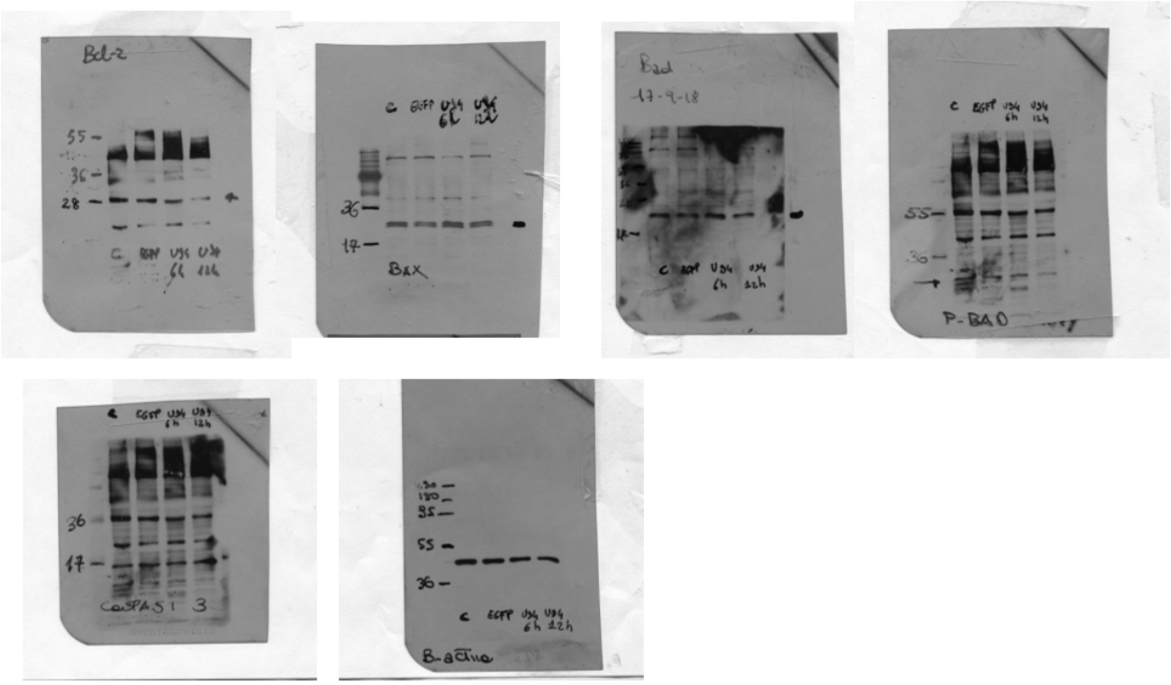

Figure S1. Immunoblots of Bcl-2, Bax, Bad, pBad, Cleaved Caspase 3 and Actin protein levels in MDA-MB-231 cells.

Figure 6

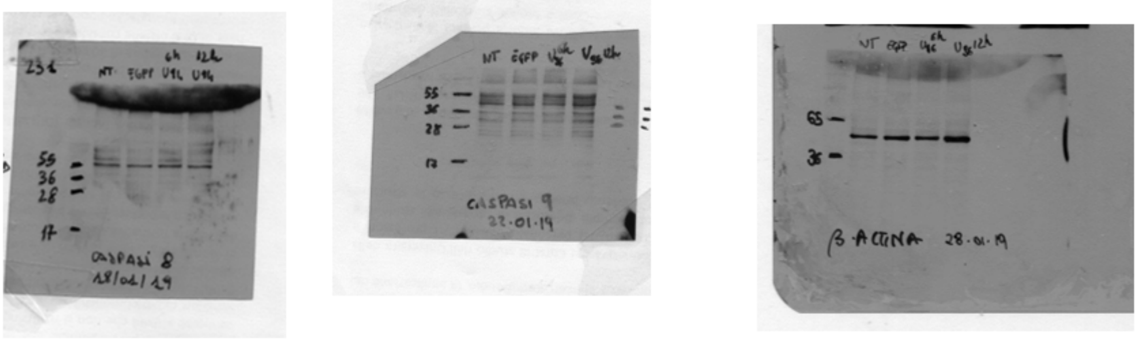

Figure S2. Immunoblots of Caspase 8, Cleaved Caspase 8, Caspase 9, Cleaved Caspase 9 and Actin protein levels in MDA-MB-231 cells.

Figure 7

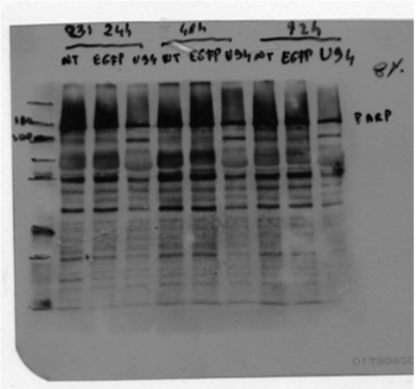

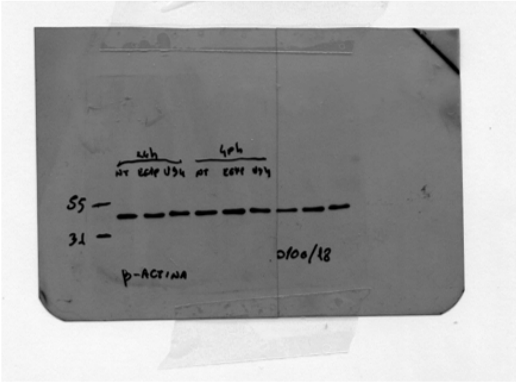

Figure S3. Immunoblots of PARP and Actin protein levels in MDA-MB-231 cells.

Figure 5

Bcl-2

NT	EGFP	6h	12h
1	0,96110338	0,5084393	0,64792486
1	1,03297933	0,68279364	0,50841497
1	0,887215	0,6357	0,583212

Bax

NT	EGFP	6h	12h
1	0,77747511	1,53556489	1,51969333
1	0,89144076	1,56392828	1,59370475
1	0,832658	1,549299	1,55553

Bad

NT	EGFP	6h	12h
1	0,77747456	1,53556438	1,51969369
1	0,84951617	1,49593165	1,63350633
1	0,813166	1,515929	1,576079

pBad

NT	EGFP	6h	12h
1	0,74358561	0,51276018	0,20581413
1	0,75854124	0,49975483	0,29642774
1	0,750707	0,506568	0,248961

Cleaved caspase-3

NT	EGFP	6 h	12 h
1	1,12034752	1,75899214	2,28696719
1	1,5667132	1,46044393	2,52576278
1	1,312589	1,630413	2,389812

Bcl-2/Bax

NT	EGFP	6h	12h
1	1,2361854	0,33110896	0,42635237
1	1,16877507	0,43658884	0,31901453
1	1,0655215	0,4103146	0,37492816

Figure 6

Cleaved caspase-9

NT	EGFP	6h	12h
1	1,04458143	1,10432368	1,79499241
1	0,91997186	0,88487436	1,58680496
1	0,979544	0,989787	1,686333

Figure 7

Cleaved PARP

NT	EGFP	U94
1	0,94208614	2,24469569
1	1,54408526	2,47501127
1	1,230654	2,355097

24h

NT	EGFP	U94
1	0,86613968	2,30948183
1	1,53580134	2,44017187
1	1,178869	2,370514

48h

Figure S4. Densitometry readings/intensity ratio of each band.
